# LIFE COURSE TIMING OF ADVERSITY AND DIURNAL CORTISOL IN LATER LIFE

**DOI:** 10.1093/geroni/igad104.3451

**Published:** 2023-12-21

**Authors:** Nadine Sikora, Briana Mezuk, James Abelson, Jane Rafferty, Jamie Abelson, Julie Ober Allen

**Affiliations:** The University of Oklahoma, Norman, Oklahoma, United States; University of Michigan, Ann Arbor, Michigan, United States; University of Michigan, Ann Arbor, Michigan, United States; University of Michigan, Ann Arbor, Michigan, United States; University of Michigan, Ann Arbor, Michigan, United States; The University of Oklahoma, Norman, Oklahoma, United States

## Abstract

Exposure to adversity is believed to alter stress sensitive biological processes. One manifestation of this, flatter diurnal cortisol slopes, has been linked to cardiometabolic problems. Questions remain about whether the timing of adversity within the life course influences its effect on cortisol slopes in later life. Two longitudinal cohort studies (Cardiac Rehabilitation and The Experience [CREATE], Tracking Risk Identification for Adult Diabetes [TRIAD]) with identical measures were merged for analysis (merged N = 175, Mage 60.8) using multilevel modeling. Measures included retrospective self-report of childhood trauma (CTQ), major life stressors in adulthood (number and recency of stressful events, affected domains, everyday discrimination), and peak-to-bedtime cortisol slopes (≤12 collected over 15 months). Report of any moderate and severe childhood adversity was not associated with adult cortisol slopes (p>.05). When we probed specific types, emotional abuse was associated with cortisol slopes (b=.006, SE=.003, p=.015). For adult adversity, total number of stressful events (b=.001, SE=.0002, p=.009) and recent stressful events (b=.003, SE=.001, p=.041) were associated with cortisol slopes. When simultaneously modeling all measures of adversity over the life course, number of stressful events in adulthood (b=. 001, SE=.0002, p=.018) and childhood emotional abuse (b=.007, SE=.004, p=.064) were the strongest predictors of flatter diurnal cortisol slopes in adulthood. Given that those reporting childhood adversity were also more likely to have experienced adulthood adversity (r=.08-.39), early life adversity may be an important risk factor for flattened cortisol slopes and associated cardiometabolic outcomes in later life via multiple pathways. Implications for intervention will be discussed.

